# ENT‐1‐Targeted Polymersomes to Enhance the Efficacy of Methotrexate in Choriocarcinoma Treatment

**DOI:** 10.1002/smsc.202400361

**Published:** 2025-01-28

**Authors:** Babak Mamnoon, Ana Paula Mesquita Souza, Tetiana Korzun, Maureen K. Baldwin, K. Shitaljit Sharma, Oleh Taratula, Yoon Tae Goo, Prem Singh, Vladislav Grigoriev, Aryan Lakhanpal, Olena R. Taratula

**Affiliations:** ^1^ Department of Pharmaceutical Sciences College of Pharmacy Oregon State University 2730 S Moody Avenue Portland OR 97201 USA; ^2^ Department of Obstetrics and Gynecology School of Medicine Oregon Health & Science University 3181 SW Sam Jackson Park Road Portland OR 97239 USA

**Keywords:** choriocarcinoma, ENT‐1‐targeted drug delivery, methotrexate, nanoplatform, polymersome

## Abstract

Gestational choriocarcinoma (CC) is a rare and highly malignant cancer originating from the trophoblastic layers of the placenta. Currently, methotrexate (MTX) is the first‐line treatment for CC; however, due to the aggressive and metastatic nature of CC, multiple doses are often required, leading to severe side effects from the lack of tumor specificity. The first targeted MTX‐loaded polymersomes (Ps) designed for efficient drug delivery to CC tumors are introduced. The modification of these Ps nanoplatforms with guanosine (Gn), which targets the ENT‐1 transporter overexpressed in CC cells, significantly enhances tumor uptake. Upon internalization by CC cells, the disulfide bonds in the Ps are reduced by high intracellular glutathione levels, causing Ps disintegration and efficient drug release. Biodistribution studies also reveal significant accumulation in subcutaneous CC tumors with minimal distribution in major organs. The ENT‐1‐targetedpolymersomes show twice the tumor accumulation compared to the nontargeted ones based on in‐vivo fluorescence imaging. ENT‐1‐targeted MTX‐loaded polymersomes (Gn‐MTX@SS‐Ps) achieve significantly greater tumor shrinkage in mice, reducing tumors by 30% more than nontargeted MTX@SS‐Ps and 75% more than free MTX at the same dosage regimen. Consequently, developed CC‐targeted MTX‐loaded polymer‐based delivery system holds the potential to significantly enhance the treatment of CC.

## Graphical abstract

ENT‐1‐targeted stimuli‐responsive polymersomes enhance the delivery and release of MTX, as demonstrated in choriocarcinoma tumors mouse model. These targeted nanocarriers improve tumor uptake and efficiently release the drug within the tumor, highlighting their potential for enhanced chemotherapy in choriocarcinoma treatment.

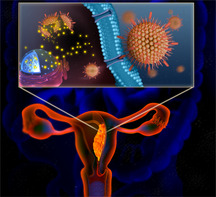

## 1 Introduction

Gestational trophoblastic disease (GTD) is benign abnormally proliferated trophoblastic tissue, such as hydatidiform molar pregnancy. GTD can become invasive and transform into malignant gestational trophoblastic neoplasia (GTN).^[^ ^1^ ^]^ GTN encompasses a spectrum of conditions, including choriocarcinoma (CC), placental‐site trophoblastic tumors, invasive moles, and epithelioid trophoblastic tumors, and it can arise from molar, ectopic, and term pregnancies, miscarriage, or abortion.^[^ ^2^, ^3^ ^]^ CC, the most common malignant GTN, is distinguished by trophoblastic hyperplasia and anaplasia, significant hemorrhage and necrosis, and the absence of chorionic villi.^[^ ^4^ ^]^ CC is a highly aggressive form of trophoblastic cancer and has a significant incidence in the United States, with 2 to 7 cases reported per 100 000 pregnancies.^[^ ^5^, ^6^ ^]^ Although CC responds to chemotherapy, the five‐year overall survival rate ranges from 82 to 92%.^[^ ^7^, ^8^ ^]^ Currently, diagnosis of CC relies on tumor biopsy, conducting histological evaluations, and blood tests to monitor serum levels of human chorionic gonadotropin (hCG).^[^ ^9^ ^]^ Gestational CC, which is the most aggressive form of trophoblast cancer, encompasses 5% of all GTN cases.^[^ ^10^ ^]^ As a form of malignant neoplasia, gestational CC readily metastasizes via hematogenous spread to various organs, such as the lungs, kidneys, brain, liver, breasts, bones, gastrointestinal tract, and lymph nodes.^[^ ^11^, ^12^ ^]^

CC treatment varies based on disease severity, ranging from a single dose of methotrexate (MTX) or actinomycin‐D (Act‐D) for low‐risk cases to a comprehensive regimen of multi‐dose chemotherapy, radiation, and surgery for high‐risk metastatic instances.^[^ ^13^ ^]^ Due to the aggressive and metastatic nature of CC, treatment frequently requires multiple doses of MTX. MTX is administered at a dosage range of 30–50 mg m^−^^2^, typically on a weekly basis, with a monotherapy success rate ranging between 57 and 90%.^[^ ^13^ ^]^ While MTX is favored for CC treatment, its poor tumor specificity in standard applications can cause severe side effects such as liver and kidney toxicity, particularly in multi‐dose chemotherapy for metastatic CC.^[^ ^14^ ^]^ Given MTX's role as the mainstay treatment for CC, the critical goal now is to enhance its effectiveness, including faster response times, while simultaneously minimizing side effects. Therefore, our study is focused on creating nanomedicines tailored for CC treatment, employing a specially designed nanoplatform that ensures precise drug delivery and release directly into CC tumors.

**Figure 1:**
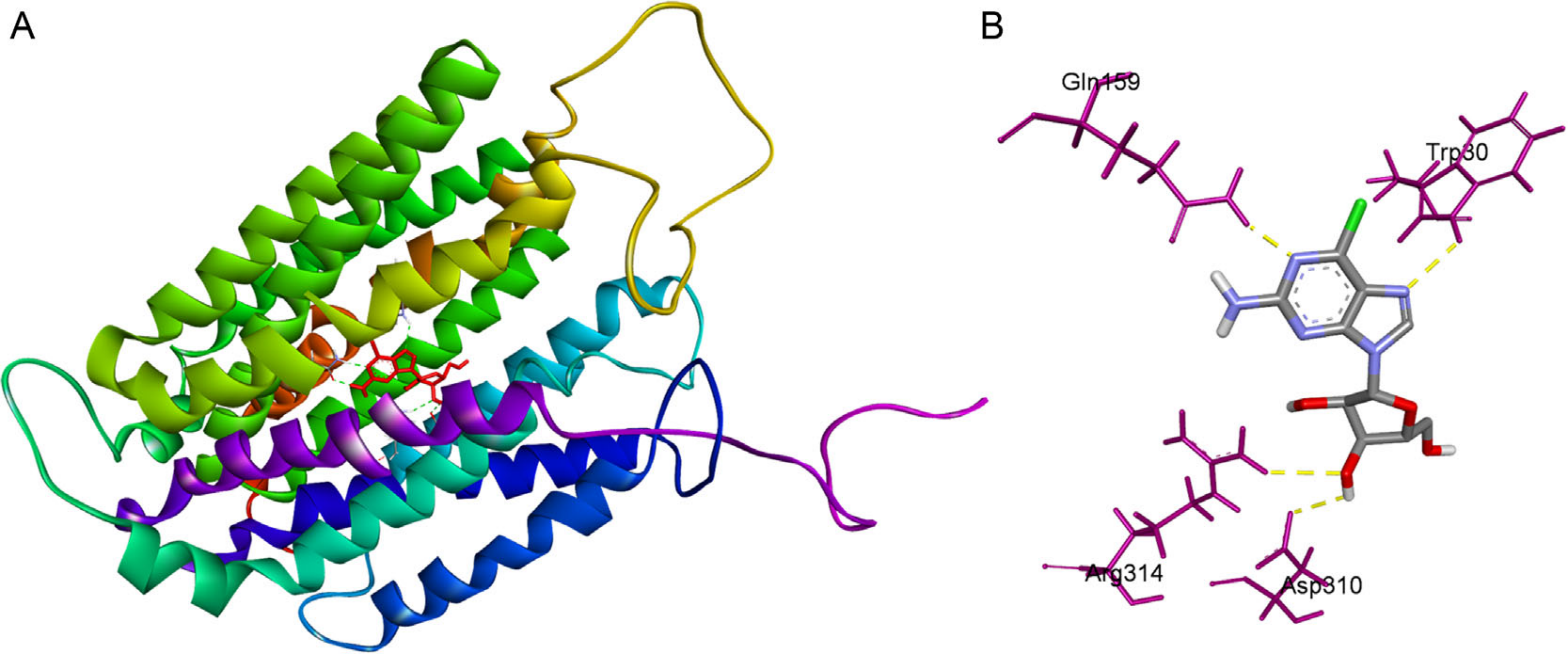
Computational ENT‐1 docking analysis of 6‐chloro‐guanosine (Gn) ligand A) within the ENT‐1 protein binding cavity and B) interacting with molecules of ENT‐1 protein. Guanosine makes 4 hydrogen bonds with Gln159, Trp 30, Arg314, and Asp310 residues within the binding cavity of ENT‐1.

We present a nanomedicine approach, providing proof‐of‐concept in a mouse model, aimed at enhancing the management of CC. We developed an MTX‐loaded polymersome (Ps) nanoplatforms functionalized with guanosine (Gn) to target the equilibrative nucleoside transporter 1 (ENT‐1) (**Figure** **1**), which is overexpressed in JEG‐3 choriocarcinoma cells, to improve CC tumor accumulation. In addition, the incorporation of a stimuli‐responsive linker ensures selective release of MTX within the intratumoral environment of the CC. We hypothesize that delivering MTX within biocompatible Ps nanocarriers, equipped with triggered intracellular release linkages and targeting ligands for CC cells, could make MTX act faster and more efficiently against CC tumors. To the best of our knowledge, our work presents the first instance of using nanoscale Ps formulation for targeted drug delivery to CC tumors.

## 2 Results and Discussion

### 2.1 Development of Guanosine (Gn)‐Modified Polymersomes

Our goal is to reduce the required dose and, thus, side effects of MTX, thereby improving therapeutic efficacy of MTX, particularly for CC treatment. Recently, our research showcased that a nano‐Ps formulation loaded with MTX successfully delivered and released MTX directly to placental cells at the implantation site in mice.^[^ ^15^ ^]^ This delivery approach required a dose six times lower than free MTX to achieve the same level of therapeutic efficacy.^[^ ^15^ ^]^ Ps, which are self‐assembled bilayer polymeric vesicles made of amphiphilic di‐block copolymers,^[^ ^16^ ^]^ are capable of encapsulating water‐soluble molecules, including MTX sodium salt, in their hydrophilic core and lipid‐soluble molecules, including contrast agents or majority of small molecule drugs, in their hydrophobic bilayer.^[^ ^17^, ^18^, ^19^, ^20^, ^21^, ^22^ ^]^ Here, we synthesized MTX‐loaded Ps formulation employing microfluidic technology (NanoAssemblr^TM^ Ignite^TM^) to generate stable and uniform polymersomes.^[^ ^23^, ^24^ ^]^ Ps were crafted using an amphiphilic di‐block co‐polymer, featuring a hydrophobic PCL (5 k) block to ensure bilayer stability and a PEG (2 k) block to establish a hydrophilic core and a water‐soluble shell. Aiming to achieve rapid release of MTX, we used Ps made from an amphiphilic copolymer containing a disulfide bond between PEG and PCL blocks (PEG‐SS‐PCL) to enable selective release of drug cargo inside cells (Figure S1, Supporting Information). Once inside CC cells, the polymersome's internal disulfide bond would undergo rapid reduction due to the elevated concentration of glutathione (GSH) within the intracellular environment. This process triggers a disulfide exchange reaction with GSH, leading to the breakdown of the nanocarriers and the efficient release of the drug.^[^ ^25^, ^26^ ^]^

To improve the delivery of nanocarrier‐loaded MTX to CC tissue, ENT‐1 was identified as an effective target for guiding the developed drug delivery system to CC cells. ENT‐1, one of the membrane transporters in human placenta, plays a crucial role in supplying nutrients and transporting small drug molecules^[^ ^27^, ^28^, ^29^ ^]^ and is overexpressed in CC cells (JEG‐3).^[^ ^30^ ^]^ ENT‐1 transports nucleosides, such as adenosine, suggesting that functionalizing the developed MTX‐loaded Ps with adenosine derivatives can enhance its delivery to CC cells with the help of the ENT‐1 transporter.^[^ ^31^ ^]^ To identify the optimal targeting ligand for the ENT1 transporter, we evaluated 20 adenosine derivatives from PubChem database^[^ ^32^ ^]^ to identify the derivative with the highest binding affinity (Table S2, Supporting Information). The ENT‐1 crystal structure was obtained from Protein Data Bank (PDB, ID: 6OB7).^[^ ^33^ ^]^ Next, to identify the derivative with the highest binding affinity toward ENT‐1, a molecular docking analysis was performed using AutoDock Vina^[^ ^34^ ^]^ to evaluate binding free energy between the ligand and target protein. The most negative binding free energy (Δ*G* < 0) of a system is the best indicator of a stable ligand–protein complex.^[^ ^35^ ^]^ As a result, 6‐chloro‐guanosine (Gn), with a binding affinity of ‐8.4 kcal mol^−1^, was selected as an optimal ligand to bind to ENT‐1 (Table S2, Supporting Information). The protein–ligand complex was visualized by the Biovia Discovery Studio Visualizer (Figure 1),^[^ ^36^ ^]^ demonstrating that Gn makes 4 hydrogen bonds with glutamine 159, tryptophan 30, arginine 314, and aspartate 310 residues within the binding cavity of ENT‐1.

**Figure 2:**
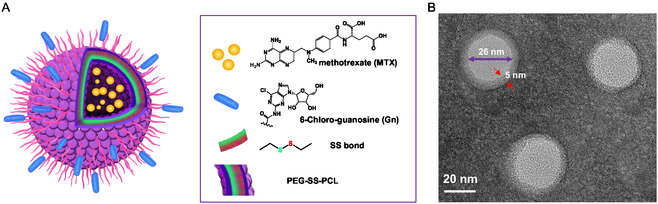
A) Schematic illustration, and B) TEM of methotrexate‐encapsulated Gn‐targeted (Gn‐MTX@SS‐Ps) polymersome.

To functionalize the Ps with Gn, the carboxylic group of PCL‐SS‐PEG (PCL‐SS‐PEG‐COOH) was conjugated to the amine group of Gn, generating a peptide bond (Figure S2–S4, Supporting Information). The conjugation yield of this reaction was ≈99%, as determined by the Pierce Quantitative Fluorometric Peptide Assay kit. To formulate Gn‐modified Ps loaded with the MTX (Gn‐MTX@SS‐Ps, **Figure** **2**A), a mixture of the prepared Gn‐PEG‐SS‐PCL and methoxy PEG‐SS‐PCL (CH_3_O‐PEG‐SS‐PCL) was used at a 1:9 ratio. Non‐targeted Ps (MTX@SS‐Ps), formulated using only CH_3_O‐PEG‐SS‐PCL without the targeting moiety, was prepared as a control (Table S1, Supporting Information). The constructed Gn‐MTX@SS‐Ps (Figure 2A) has a spherical shape (Figure 2B), nearly neutral surface charge (−2.48 ± 0.21, *n* = 3), uniform distribution (PDI of 0.11), and a hydrodynamic size of ≈42 nm (Table S1, Supporting Information). Transmission electron microscopy confirms the Gn‐MTX@SS‐Ps's double‐layer structure with ≈26 nm in diameter of the inner hydrophilic sphere and ≈5 nm outer hydrophobic layer (Figure 2B). These Ps allowed loading 30 mg of MTX per 10 mg of polymer in 1 mL of solution, for both non‐targeted and targeted polymersomes. Previously, we confirmed that MTX@SS‐Ps effectively releases MTX in solution, exceeding 80% drug (MTX) release at 1 mM GSH. Since cytosolic concentrations of GSH are between 1 and 10 mM,^[^ ^37^ ^]^ the high MTX release observed from MTX@SS‐Ps at 1 mM GSH is promising. Storing Gn‐modified MTX‐loaded polymersomes at 4 °C for 4 weeks resulted in only minimal size changes (Figure S5, Supporting Information), indicating good stability and extended shelf life for this formulation.

### 2.2 Evaluation of ENT‐1‐Targeted Gn‐modified Polymersomes in vivo

**Figure 3:**
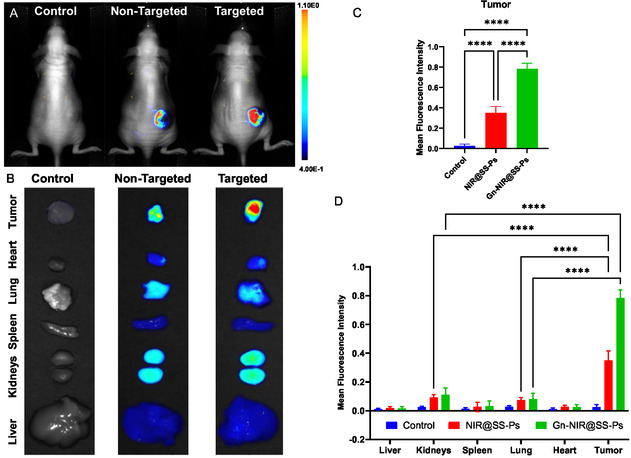
Biodistribution of NIR dye‐loaded non‐targeted and targeted NIR@SS‐Ps. Fluorescence images of mice bearing JEG‐3 subcutaneous tumors A) and resected major organs/tumors B) mice (*n* = 3), 12 h post‐injection (i.v.) of saline (control), NIR@SS‐Ps (non‐targeted, 0.3 mg mL^−1^ dye, 100 μL) and Gn‐NIR@SS‐Ps (targeted, 0.3 mg mL^−1^ dye, 100 μL). Semiquantitative analysis of fluorescence signal in tumors and organs C,D), 12 h post‐injection with NIR@SS‐Ps, Gn‐NIR@SS‐Ps, and saline (control). Values are expressed as mean ± SD, *n* = 3. (C)– assessed with one‐way ANOVA, (D)– assessed with two‐way ANOVA * *p* < 0.05, **p < 0.01, ***p < 0.001, ****p < 0.0001.

To determine if Gn modification enhances the uptake of Ps by CC tumors in vivo, we evaluated the biodistribution of non‐targeted and ENT‐1‐targeted Ps using fluorescence imaging. This was achieved by monitoring the fluorescence of the loaded NIR dye (Figure S6, Supporting Information). At 12 h post‐injection (i.versus.), both NIR@SS‐Ps and Gn‐NIR@SS‐Ps produce a prominent fluorescence signal in subcutaneous JEG‐3 tumors (**Figure** **3**A,B). The ENT‐1‐targeted polymersome outperformed the non‐targeted ones in terms of tumor accumulation (Figure 3C), as evidenced by the fluorescence intensity that was twofold greater in the tumors treated with the targeted Ps (Gn‐NIR@SS‐Ps) in comparison with the non‐targeted group (NIR@SS‐Ps). Fluorescence imaging of resected organs revealed near complete clearance of NIR@SS‐Ps and Gn‐NIR@SS‐Ps from major organs (Figure 3A,B). While some accumulation of Ps was observed in kidneys and lungs, the signal was comparable to background levels, and there was no significant difference in fluorescence intensity between these organs and those of control mice administered saline (Figure 3D). The fluorescence signal of non‐targeted Ps in kidneys was 25% of that observed in tumors. Furthermore, the difference was even more prominent with the Gn‐NIR@SS‐Ps, with an almost sevenfold (86%) increase in fluorescence intensity in tumors compared to kidneys (Figure 3D). These biodistribution data indicate a preferential uptake and retention of the Ps in the CC tumors enhanced by ENT‐1‐targeting, suggesting therapeutic promise for improved antineoplastic efficacy and substantially minimized off‐target side effects.

**Figure 4:**
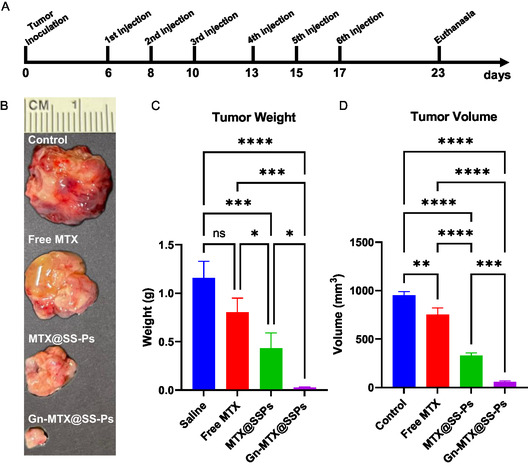
ENT‐1‐targeted MTX‐based treatment of CC. A) Schematic timeline of targeted anticancer therapy performed in mice 6 days following subcutaneous tumor inoculation. Free MTX, MTX@SS‐Ps, Gn‐MTx@SS‐Ps, and saline as control were injected every 2^nd^ day (10 mg kg^−1^, 6 doses total, i.v.), followed by tumor growth monitoring for 6 days (*n* = 3). Photographs B) and quantitative analysis of weights C) and volume D) of JEG‐3 subcutaneous tumors resected from mice at day 23 after treatment with 6 doses of free MTX, MTX@SS‐Ps, Gn‐MTX@SS‐Ps, and saline control. (C and D) are expressed as mean ± SD, *n* = 3, assessed with one‐way ANOVA, *p < 0.05, **p < 0.01, ***p < 0.001, ****p < 0.0001.

Given the promising in vivo biodistribution data, we further evaluated the antitumor activity of ENT1‐targeted Gn‐MTX@SS‐Ps in a JEG‐3 tumor mouse model. When subcutaneous tumors reached ≈100 mm^3^ in volume, the mice were treated with Gn‐MTX@SS‐Ps and controls (free MTX, MTX@SS‐Ps, and saline) every other day (10 mg kg^−1^, for a total of 6 doses, **Figure** **4**A). Tumors in both treated and control mice were monitored for an additional six days following the last injection. Throughout the 23‐day study period, using a consistent dose regimen (10 mg kg^−1^ MTX per dose), tumors in the saline and free MTX groups continued to grow, whereas the non‐targeted MTX@SS‐Ps led to a deceleration of tumor progression. Importantly, the targeted Gn‐MTX@SS‐Ps halted tumor growth (Figure S7, Supporting Information). After euthanasia, the tumor volumes, along with tumor weight, in the Gn‐MTX@SS‐Ps treatment group were significantly reduced, by 95% compared to the non‐treated (saline) group (Figure 4B–D). Tumor growth halted after the 2^nd^ dose and continued to decrease with subsequent doses in the Gn‐MTX@SS‐Ps treatment group, whereas tumors in other groups continued to grow at varying rates (Figure 4D). In comparison, the non‐targeted MTX@SS‐Ps reduced tumor growth by 65%, and free MTX by only 20%, relative to the saline control group. The tumors treated with Gn‐MTX@SS‐Ps exhibited the most potent treatment effect, demonstrating ≈6‐fold higher antitumor efficacy compared to the non‐targeted MTX@SS‐Ps.

The promising antitumor efficacy was primarily due to the enhanced cellular uptake of ENT‐1‐targeted polymersomes and the effective release of MTX within the tumor, facilitated by efficient intracellular disulfide bond cleavage in the presence of GSH. These findings underscore the clear advantage of ENT1 targeting and tumor‐sensitive drug release in chemotherapy for the treatment of CC.

**Figure 5:**
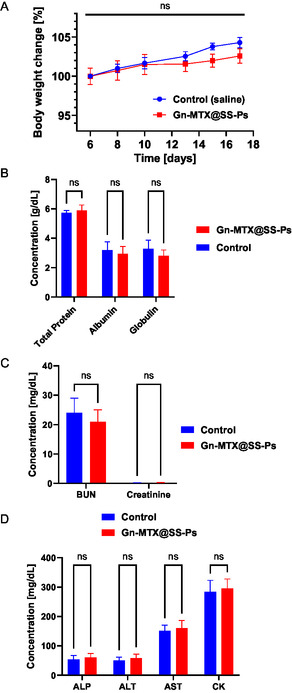
Safety evaluation of ENT‐1‐targeted MTX‐loaded polymersomes. The body weights A) and blood levels of total protein, albumin, and globulin B), BUN and creatinine, illustrating kidney function C), and CK, illustrating cardiac function, and ALT, ALP, and AST, illustrating liver function D) of mice following treatment with targeted Gn‐MTX@SS‐Ps vs. non‐treated mice (saline control). (B,C) are expressed as mean ± SD, *n* = 3, assessed with two‐way ANOVA.

The mice treated with Gn‐MTX@SS‐Ps showed no signs of toxicity, weight loss, or mortality throughout the study (**Figure** **5**A). Blood chemistry analysis further revealed no significant differences in serum levels of blood urea nitrogen (BUN), alkaline phosphatase (ALP), alanine aminotransferase (ALT), aspartate transaminase (AST), creatine kinase (CK), proteins, and electrolytes among animals after 6 doses (10 mg kg^−1^) of targeted Gn‐MTX@SS‐Ps (Figure 5B–D). Therefore, the ENT‐1 targeting moiety does not compromise the safety profile of the polymersome.

## 3 Conclusion

This study introduces MTX‐loaded polymersomes (Ps) targeted to the ENT‐1 transporter overexpressed in choriocarcinoma cells, aiming to enhance treatment efficacy of MTX therapy and minimize systemic side effects. Functionalizing Ps with guanosine (Gn) significantly improved cellular uptake and efficient drug release within the tumor, driven by intracellular disulfide bond cleavage in the presence of GSH. In vivo fluorescence imaging showed substantial accumulation of targeted Ps in CC tumors with minimal distribution in major organs. In a mouse model, ENT‐1‐targeted Ps demonstrated a 95% reduction in tumor volume, exhibiting ≈six‐fold higher antitumor efficacy compared to non‐targeted Ps. The study highlights the potential of ENT‐1‐targeted polymersomes for improved chemotherapy in choriocarcinoma treatment, with enhanced drug delivery and reduced systemic toxicity. The study successfully demonstrated that ENT‐1‐targeted MTX‐loaded polymersomes significantly improve drug delivery and antitumor efficacy in JEG‐3 mouse model, for application in choriocarcinoma treatment.

## 4 Experimental Section

4.1

4.1.1

##### Materials

PEG(2 k)‐SS‐PCL(5 k) (methoxy poly (ethylene glycol)‐b‐disulfide‐poly(ε‐caprolactone) copolymer was purchased by Ruixibiotech (Xian, China). NIR dye (silicon 2,3‐naphthalocyanine bis (trihexylsilyloxide)) was purchased from Alfa Chemistry (Ronkonkoma, NY, USA). USP‐grade methotrexate sodium salt (MTX) was obtained from OHSU Pharmacy (Hikma Pharmaceuticals USA Inc., Berkeley Heights, NJ). 2‐Amino‐6‐chloropurine riboside (6‐chloro‐guanosine) was purchased from TCI America (Portland, OR). Common chemicals and supplies were provided by MilliporeSigma (Milwaukee, WI), Fisher Scientific Inc. (Hampton, NH), and VWR International, LLC (Radnor, PA).

##### Computational Evaluation of Targeted Moieties for the Choriocarcinoma Cell ENT‐1 Transporter

To identify the best ligand for binding to the ENT‐1 transporter, a group of 20 adenosine analogs from the PubChem database was selected.^[^ ^32^ ^]^ The ENT‐1 crystal structure was generated by the Protein Data Bank (PDB, ID: 6OB7),^[^ ^33^ ^]^ followed by conducting a molecular docking study using AutoDock Vina.^[^ ^34^ ^]^ The structure of the ligand molecules was prepared based on their appropriate 3D conformation, while their energy was minimized using PyRx^[^ ^38^ ^]^ and optimized using Chimera software version 1.17.3.^[^ ^39^ ^]^ and Modeller v.10.4 to fix the missing residues of the ENT‐1 protein transporter.^[^ ^40^ ^]^ The protein–ligand complex was visualized by the Biovia Discovery Studio Visualizer.^[^ ^36^ ^]^

##### PEG(2 k)‐SS‐PCL(5 k) Modification with Guanosine (Gn)

PCL‐SS‐PEG‐COOH polymer (100 mg, 14 μmol, 1 equiv.) dissolved in dimethylformamide (5 mL), 6‐chloro‐guanosine (5 mg, 17 μmol, 1.2 equiv.) was added. After stirring for 10 min, triethylamine (200 μL) and EDC (4 mg, 26 μmol, 1. 8 equiv.) were added to the reaction mixture and kept overnight stirring. Next, the reaction mixture was diluted with dichloromethane (25 mL), transferred to a separating funnel, and washed with saturated saline solution three times. The organic layer was collected, dried using anhydrous sodium sulfate, and concentrated using a rotavap. The residue solution was added to chilled ether (−20 °C, 45 mL), and the precipitate was collected by centrifugation at 4 °C. Trace ether present on the precipitate was removed under vacuum at room temperature. To further remove any impurity, the obtained polymer was dissolved in tetrahydrofuran (THF, 2 mL), loaded on a size exclusion column (Bio‐Beads S‐X1, Cat. No. 52‐2150) and eluted with THF under gravity. The fractions were collected, concentrated under rotavap, precipitated out in chilled ether (−20 °C), dried the precipitate after collecting by centrifugation, and characterized by 1H NMR. Yield: 98 mg (94%). 1H NMR (CDCl_3_, 400 MHz): δ 8.08–8.11 (m, 2H), δ 6.66–6.67 (t, 2H), δ 5.51–5.53 (m, 8H), δ 4.16–4.30 (m, 6H), δ 3.93–4.05 (m, 94H), δ 3.87–3.99 (m, 22H), δ 3.54−3.57 (m, 181H), δ 3.17 (s, 6H), δ 3.07 (s, 2H), δ 2.74–2.92 (m, 10H), δ 2.20–2.31 (m, 96H), δ 1.53–1.65 (m, 192H), δ 1.27–1.35 (m, 94H). The conjugation of PCL‐SS‐PEG‐COOH with 6‐chloro‐guanosine, which exists as α‐form and β‐form (anomers), leads to the formation of a mixture of two isomeric products. This has been confirmed by the observation of two protons in the NMR peaks that correspond to that of conjugated 6‐chloro‐guanosine (Figure S2–S4, Supporting Information).

To evaluate the conjugation yield of Gn to polymer, the Pierce Quantitative Fluorometric Peptide Assay kit (Thermo Scientific, Rockford, IL) was used to measure the free amine groups before filtering the reaction mixture based on the manufacturer's recommendation. According to the amount of the initial polymer used for the polymersome preparation, the yield of Gn‐conjugated PEG‐SS‐PCL was determined to be 99%.

##### Polymersome Preparation

MTX‐loaded polymersomes were prepared using the NanoAssemblr Ignite microfluidic mixer (Precision NanoSystems). First, methotrexate sodium salt (30 mg) was dissolved in 1 mL saline (syringe 1). To prepare non‐targeted nanoparticles, PEG‐SS‐PCL polymer (M.W. = 7 kDa, 10 mg, RuixiBiotech, China) was added to 1 mL acetone (syringe 2). Targeted polymersome samples were prepared using 9 mg of the PEG‐SS‐PCL polymer and 1 mg of the conjugated PCL‐SS‐PEG‐Gn polymer (10%) in 1 mL acetone (syringe 2). The syringes were installed into the NanoAssemblr Ignite Ignite platform through a NxGen cartridge (Precision Nanosystems). The organic and aqueous solutions were run at 1:1 flow ratio followed by 9 mL min^−1^ total flow rate. The polymersome sample mixture was then collected in a glass vial and bubbled air to remove the organic phase. Finally, the sample was centrifuged using an Amicon Ultra‐4 centrifugal filter tube (MWCO: 30 kDa, 10 min), and the top solution was collected and passed through 0.2‐micron filter. For NIR Imaging, NIR dye (naphthalocyanine derivative) was loaded into polymersomes using a glass microfluidic mixer chip (Precigenome, San Jose, CA) compatible with THF: copolymer (10.0 mg, PEG‐SS‐PCL) and NIR dye (0.3 mg) were dissolved in tetrahydrofuran (1 mL total) to generate NIR‐SS‐Ps.

##### Characterization of Nanoparticles

The polymersomes’ size, surface charge, and polydispersity were determined using a Malvern ZetaSizer (Worcestershire, UK). Transmission electron microscopy images were acquired on JEOL JEM‐2100 LB6 (Peabody Massachusetts) at North Dakota State University (NDSU) Electron Microscopy Core to assess the morphology of polymersomes. This material is based upon work supported by the National Science Foundation under Grant No. 0 821 655. The loading of MTX into the polymersome nanoparticles was evaluated using high‐performance liquid chromatography (Shimadzu, Japan) at a wavelength of 302 nm. The analysis was performed with an Agilent ZORBAX C‐18 column (3.5 μm, 4.6 × 75 mm), operating at a flow rate of 1 mL min^−1^. The mobile phase consisted of acetonitrile/water (35:65 v v^−1^) with 0.1% trifluoroacetic acid.

##### Animals

All animal experiments were approved by the Institutional Animal Care and Use Committee of Oregon Health and Science University and were carried out in accordance with national and local guidelines and regulations (IP00000033). Athymic nude mice were obtained from Charles River Laboratories (Wilmington, MA).

##### Biodistribution Study

Choriocarcinoma tumor‐bearing mice (3 animals per group, *n* = 3) were injected intravenously (i.v.) via tail vein with NIR@SS‐Ps or Gn‐NIR@SS‐Ps (100 μL at 0.3 mg mL^−1^ SiNc in saline) as non‐targeted and targeted polymersomes, respectively, along with saline as the control group, and biodistribution of NIR dye loaded polymersomes was evaluated in major organs and tumors 12 h after i.v. injection, using a LI‐COR Pearl Impulse Imaging System (LI‐COR, Lincoln, NE) with an 800 nm channel. The mean fluorescence intensity of the region of interest was quantified using Pearl Impulse Software.

##### Antitumor Efficacy

Choriocarcinoma tumor‐bearing mice were divided into 4 different groups (3 animals per group, *n* = 3): control (saline), free MTX, MTX@SS‐Ps, and Gn‐MTX@SS‐Ps. The MTX‐treatment groups (free MTX, MTX@SS‐Ps, and Gn‐MTX@SS‐Ps) were administered 10 mg kg^−1^ dose of free MTX or an equivalent dose of MTX‐loaded polymersomes (MTX@SS‐Ps and Gn‐MTX@SS‐Ps) on days 6, 8, 10, 13, 15, and 17 after tumor inoculation. Length and width of tumors were measured using calipers for 10 days following treatment and used to calculate tumor volume as V = W (2) × L/2, where V, W, and L are volume, width, and length of the tumor, respectively. Mice were euthanized on day 23.

##### Safety Evaluation of MTX‐Loaded Polymersomes

In a separate study, 2 groups of mice were treated with saline (control) and MTX@SS‐Ps nanoparticles (treatment group, 10 mg kg^−1^) every other day for two weeks (total 6 injections). Body weight was recorded every other day. At the end of week 3, the mice were euthanized and their whole blood was collected and submitted to IDEXX Laboratories for a total Health Profile screen^[^ ^41^, ^42^ ^]^ to determine plasma levels of liver, renal, and cardiac function indicators, including creatinine, BUN, AST, ALP, CK, ALT, albumin, and globulin.

##### Statistical Analysis

All data processing was conducted using GraphPad Prism v10 (GraphPad Software, CA, USA) and results were expressed as the mean ± SD. The sample size (n) for each study is specified in the figure legends. A two‐tailed unpaired t‐test was used for comparisons between two groups, while one‐way analysis of variance was applied for comparisons among more than two groups. The statistical significance threshold was measured as *P* < 0.05 (*p < 0.05, **p < 0.01, ***p < 0.001, ****p < 0.0001).

## Conflict of Interest

The authors declare no conflict of interest.

## Author Contributions

**Babak Mamnoon**: investigation (equal); methodology (equal); validation (equal); and writing—original draft (equal). **Ana Paula Mesquita Souza**: data curation (equal); investigation (equal); methodology (equal); and writing—original draft (equal). **Tetiana Korzun**: data curation (equal); formal analysis (equal); software (equal); and writing—review and editing (equal). **Maureen K. Baldwin**: conceptualization (equal) and writing—review and editing (equal). **K. Shitaljit Sharma**: data curation (supporting); formal analysis (supporting); and software (supporting). **Oleh Taratula**: writing—review and editing (equal). **Yoon Tae Goo**: data curation (supporting) and methodology (supporting). **Prem Singh**: formal analysis (supporting); methodology (supporting); and validation (supporting). **Vladislav Grigoriev**: data curation (supporting) and methodology (supporting). **Aryan Lakhanpal**: data curation (supporting) and formal analysis (supporting). **Olena R. Taratula**: conceptualization (lead); investigation (lead); methodology (lead); supervision (lead); validation (lead); and writing—review and editing (lead). **Babak Mamnoon** and **Ana Paula Mesquita Souza** contributed equally to this work.

## Supporting information

Supplementary Material

## Data Availability

The data that support the findings of this study are available from the corresponding author upon reasonable request.
