# Supplementary material for: ENT‐1‐Targeted Polymersomes to Enhance the Efficacy of Methotrexate in Choriocarcinoma Treatment
Source: Small Sci. 2025 Jan 28;5(7):2400361. doi: 10.1002/smsc.202400361 (PMC12257878; doi:10.1002/smsc.202400361)
Supplement: Supplementary file 1 — Supplementary Material [file SMSC-5-2400361-s001.pdf]

Supporting Information

ENT-1 Targeted Polymersomes to Enhance the Efficacy of Methotrexate in Choriocarcinoma Treatment

*Babak Mamnoon, Ana Paula Mesquita Souza, Tetiana Korzun, Maureen K. Baldwin, K. Shitaljit Sharma, Oleh Taratula, Yoon Tae Goo, Prem Singh, Vladislav Grigoriev, Aryan Lakhanpal, and Olena R. Taratula**

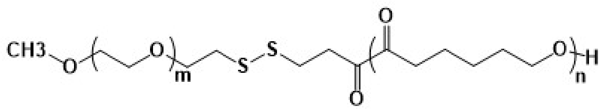

PEG(2k)-SS-PCL(5k)

Figure S1. PEG(2k)-SS-PCL(5k) (methoxy poly (ethylene glycol)-*b*-disulfide-poly(ε-caprolactone).

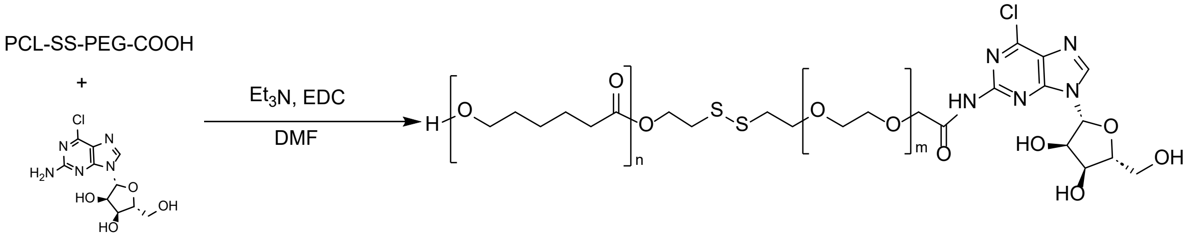

Figure S2. Conjugation of PCL-SS-PEG-COOH with 6-chloro-guanosine using EDC as the coupling agent.

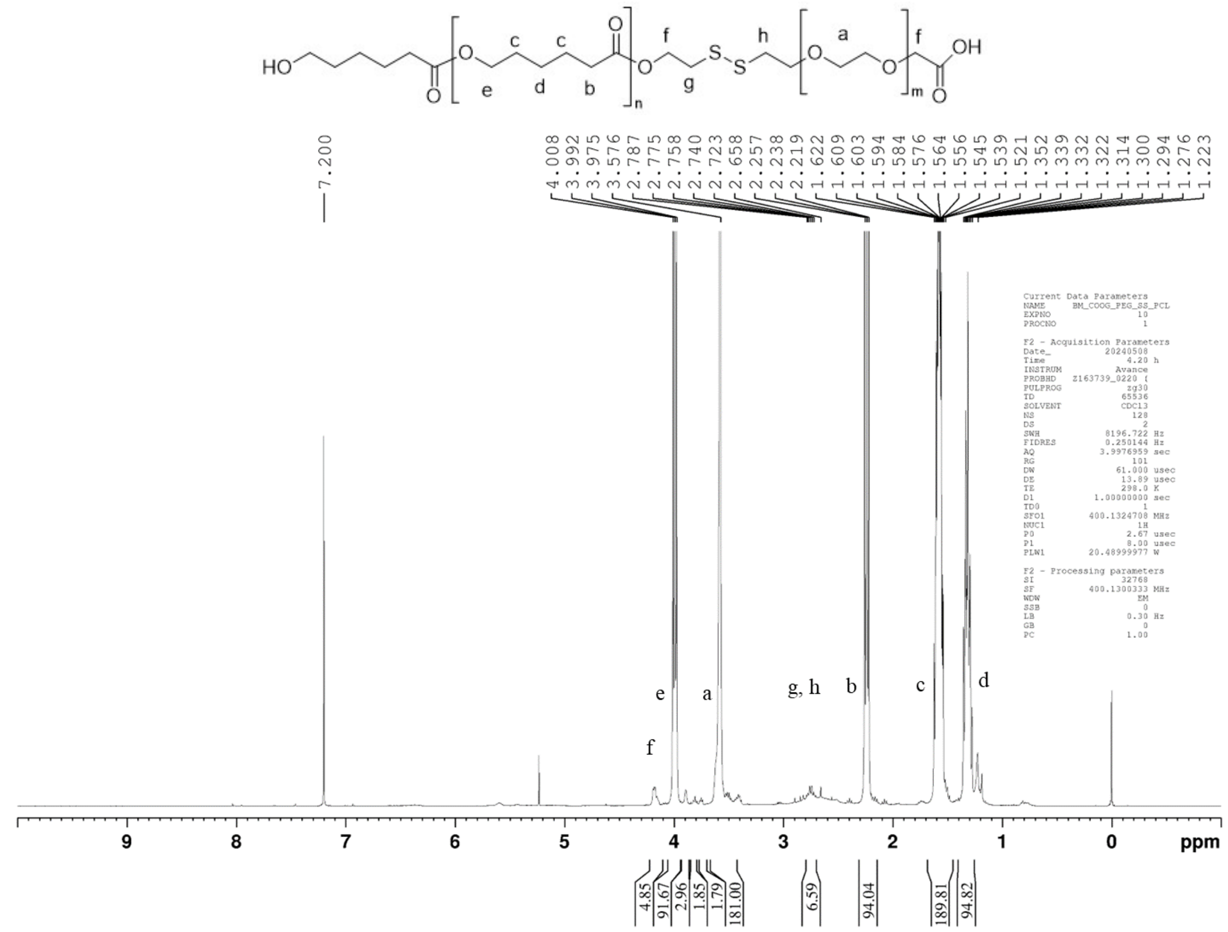

Figure S3. ^{1}H NMR ($CDCl_3$, 400 MHz) of PEG(2k)-SS-PCL(5k) (before conjugation with Gn).

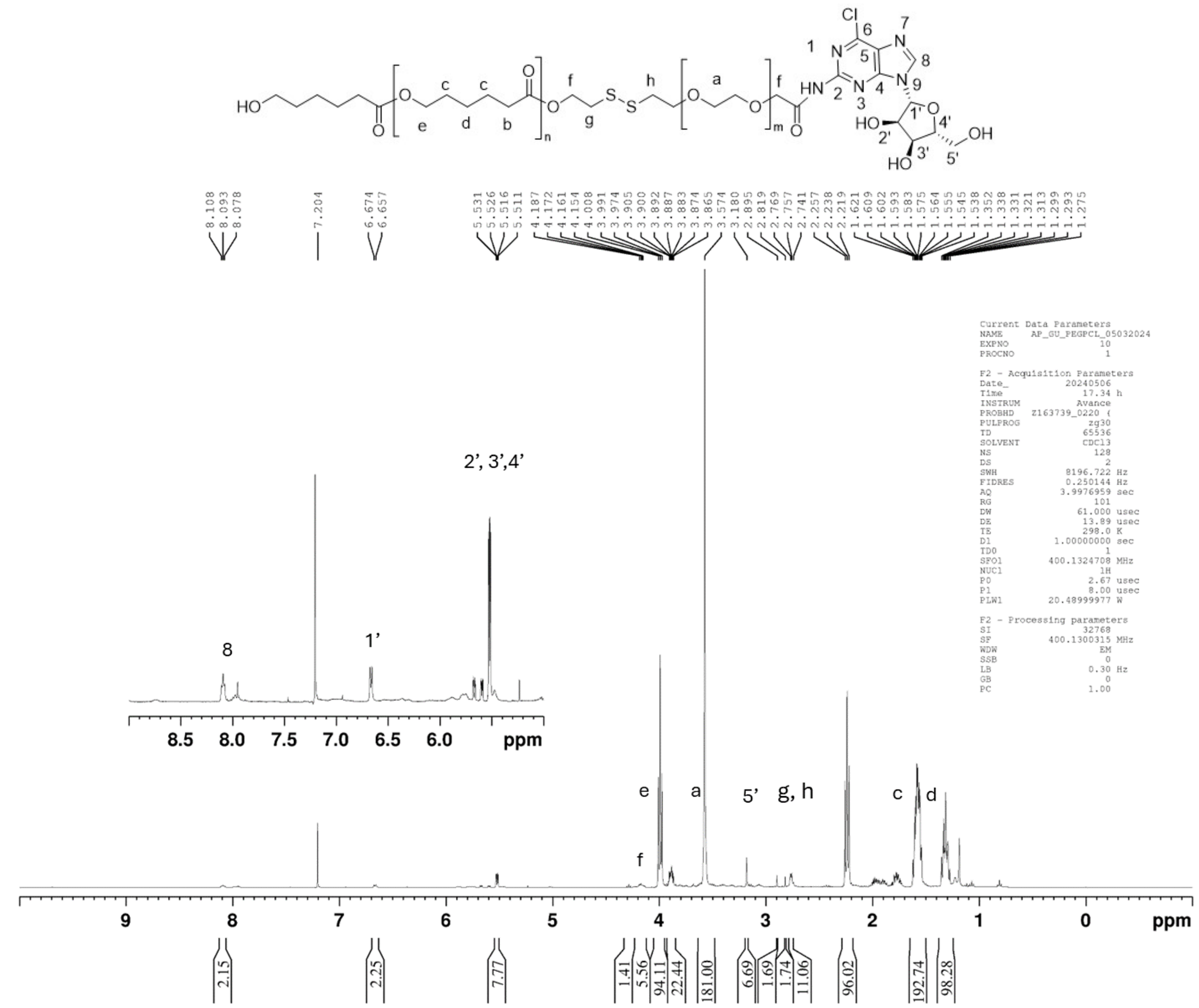

Figure S4. ^{1}H NMR ($CDCl_3$, 400 MHz) of PEG(2k)-SS-PCL(5k) conjugated with 6-chloro-guanosine (Gn) (Insert shows the magnification of 5 ppm to 9 ppm).

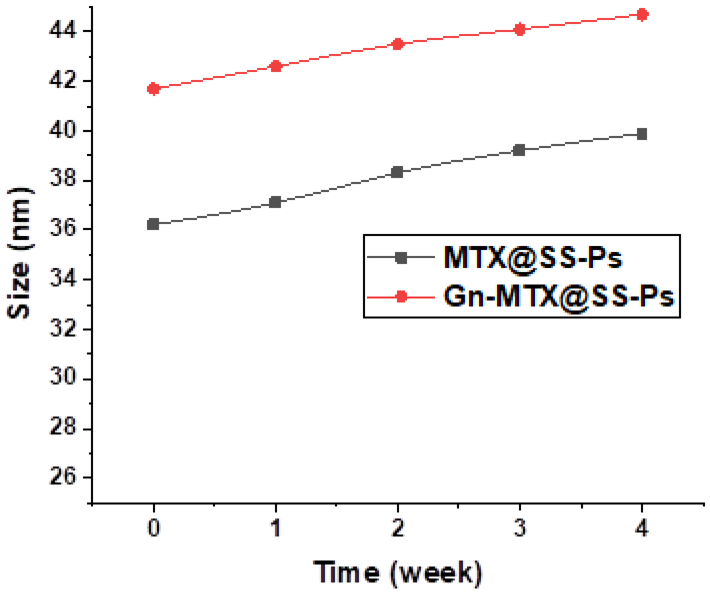

Figure S5. Stability study via DLS size measurements for MTX@SS-Ps (black) and Gn-MTX@SS-Ps (red) over 4 weeks.

Table S1. Polymersome's size (by DLS, diameter in nm ± Standard Deviation (SD)), polydispersity index (PDI) ± SD, and zeta potential (mV ± SD).

Nanoagent	Size, nm	PDI	Zeta potential, mV
MTX@SS-Ps	37.6 ± 0.3	0.10 ± 0.05	-2.25 ± 0.36
Gn-MTX@SS-Ps	42.5 ± 0.3	0.11 ± 0.03	-2.48 ± 0.21
NIR@SS-Ps	38.3 ± 0.5	0.12 ± 0.01	-1.38 ± 0.12

Table S2. Binding affinity (kcal/mol) of selected adenosine derivatives for ENT-1 protein.

Number	Ligand	Binding affinity, kcal/mol	Compound CID
1	Adenosine	-7.3	60961
2	Amino-adenosine	-8.2	72200
3	Amino-purine riboside	-8.1	11065406
4	Chloro-5-Deoxyadenosine	-7.4	5327118
5	Chloro-adenosine	-7.8	8974
6	Chloro-guanosine	-8.4	102197
7	Chloro-tubercidin	-7.8	97453

8	Cordyceptin	-7.1	6303
9	Deaza-adenosine	-7.2	23190
10	Deoxy-2-fluoroadenosine	-7.8	447864
11	Deoxy adenosine	-7.3	439182
12	Deoxy tubercidin	-7.5	3006222
13	Fluoro-5-deoxyadenosine	-7.4	100253
14	Fluoro-adenosine	-7.6	8975
15	Formycin A	-7.9	135449281
16	Guanosine	-8.0	135398635
17	Thioadenosine	-6.9	128924
18	Thioguanosine	-6.8	2724387
19	Tubercidin	-7.6	6245
20	Xylosyl-adenine	-7.3	160739

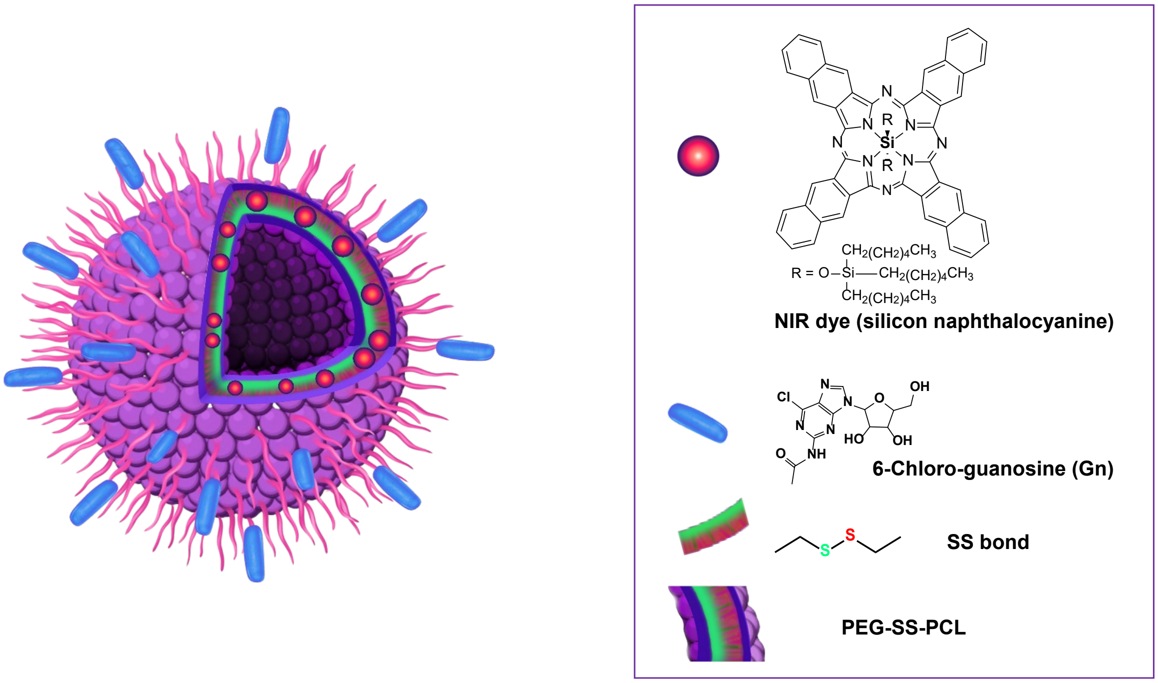

Figure S6. Schematic illustration of Gn-targeted NIR-dye-encapsulated polymersome (Gn-NIR@SS-Ps).

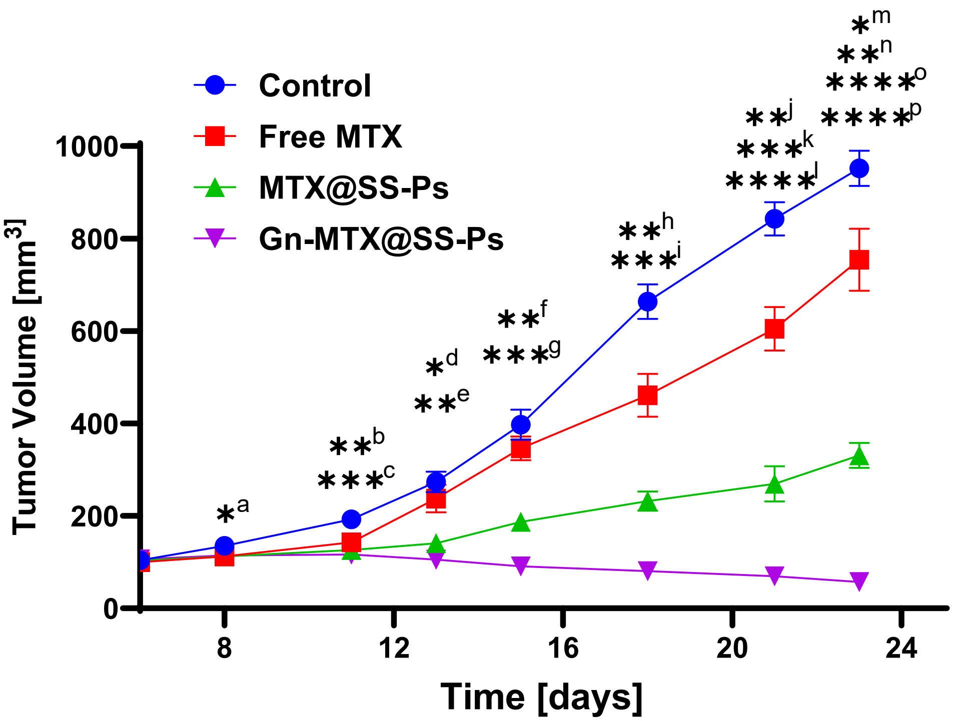

Figure S7. Growth profiles of subcutaneous JEG-3 tumors resected from mice at day 23 after treatment with 6 doses (10 mg kg^{-1}) free MTX, NIR@SS-Ps, Gn-NIR@SS-Ps, and saline control. Values are expressed as mean ±SD (n=3). *P < 0.05, **P < 0.01, ***P < 0.001, ****P < 0.0001.

a – Control vs Free MTX, Control vs. MTX@SS-Ps, and Control vs. Gn-MTX@SS-Ps; b - Control vs. Free MTX, Control vs. MTX@SS-Ps; c - Control vs. Gn-MTX@SS-Ps; d - Free MTX vs. MTX@SS-Ps and MTX@SS-Ps vs. Gn-MTX@SS-Ps; e - Control vs. Gn-MTX@SS-Ps, Free MTX vs. Gn-MTX@SS-Ps; f - Control vs. MTX@SS-Ps, Control vs. Gn-MTX@SS-Ps, Free MTX vs. MTX@SS-Ps, MTX@SS-Ps vs. Gn-MTX@SS-Ps; g - Free MTX vs. Gn-MTX@SS-Ps; h - Control vs. Free MTX, Free MTX vs. MTX@SS-Ps, Free MTX vs. Gn-MTX@SS-Ps; i - Control vs. MTX@SS-Ps, Control vs. Gn-MTX@SS-Ps, MTX@SS-Ps vs. Gn-MTX@SS-Ps; j - Control vs. Free MTX, Free MTX vs. Gn-MTX@SS-Ps, MTX@SS-Ps vs. Gn-MTX@SS-Ps; k - Control vs. Gn-MTX@SS-Ps, Free MTX vs. MTX@SS-Ps; l - Control vs. MTX@SS-Ps; m - Control vs. Free MTX; n - Free MTX vs. MTX@SS-Ps, Free MTX vs. Gn-MTX@SS-Ps; o - Control vs. MTX@SS-Ps; p - Control vs. Gn-MTX@SS-Ps.
